# Extraordinary Precocity in the Development of the Male Sexual Organs and Muscular System in a Child Four Years Old

**Published:** 1853-01

**Authors:** 


*Extraordinary Precocity in the Development of the Male Sexual Organs and Muscular System in a Child Four Years Old.* By Robert King Stone, M. D., Professor of Physiological Anatomy in the National Medical College, and one of the Surgeons of the Washington Infirmary. (Read to the Pathological Society of the District of Columbia.)

I have the honor to present to the Society one of the most extraordinary cases of precocious development of the male sexual organs and general muscular system now on record:

Mr. Charles S——, of this district, brought his son, Theodore, to my house on the 14th of September, 1852, his birthday, for my inspection and opinion; stating that on that day he was four years old. I at once declared my incredulity, for his height and robust development seemed those of a child at least six years older than the age he mentioned. My astonishment was greatly increased, when, on stripping the boy, he offered to my view the well-developed sexual organs of a man, and the pubes covered with a luxuriant growth of hair.

I was perfectly incredulous that the boy was born on the 14th of September, 1848; but his father said he could produce his certificate of nativity, and that he, with his mother, the midwife who delivered him, and fifty other responsible persons, would swear that he had stated his age correctly.

The boy is remarkably handsome, and when stripped, presents a form of great beauty, which is, in fact, a miniature model of a perfectly developed athletæ.

The condition of his muscular and osseous system is extraordinary; the deltoids and other muscles of the arm, forearm, back, and thorax, have the same relations to his height that those of a hard-laboring man would have of the stature of six feet. The muscles of the thigh, gluteal region, and leg, are perhaps better developed than those of the upper extremity, but in nearly the same ratio to the height.

If the child's face is concealed, the examiner would declare his figure to be that of a miniature man, perfectly developed, and at least twenty-one years of age.

There seems to be little adipose tissue about him, the muscular prominences being clear, and well defined, as if produced by constant exercise or hard labor.

The growth of hair is distinct in the axilla, but by no means so marked as that upon the pubes. As in very robust men, the lumbar and sacral regions are covered with a thick down of dark hair.

His height is now four feet one-quarter inch, and weight nearly seventy pounds; though his mother informs me he weighed seventy-five pounds in the spring, and attributes his diminution to the great number of lumbricoides which infest him.

His penis is that of a well-developed man, measuring in a semi-flaccid state four and a quarter inches in length, and in the state of perfect flaccidity three and a half inches. The prepuce is short, leaving exposed a perfectly formed glans penis. I might state, also, that the papillæ of the corona glandis are in a state of hypertrophy, being distinctly salient, and exquisitely sensitive. The pubes are covered with a luxuriant growth of crisp, curling, dark-brown hair, as found in the adult state. In the scrotum, presenting the appearance of the adult, are two firm, apparently well-developed testicles, perhaps rather under the average size of those organs in the adult. Independently of the penis, the development of these alone would have been decidedly remarkable at that tender age.

The spermatic cords are distinct, and under the finger give the impression of perfect organs.

Carefully examined from the neck down, the appearances are those of a *perfect man*, whilst the head and face were those of a child. On examining his mouth, it was found to contain only the twenty deciduous teeth of his age, with the exception of the middle incisors of the upper jaw, which were carious to the fangs.

The head was perfectly formed, and bears a proper proportion to the development of the body.

The breadth between the ears across the cerebellum was great; in fact, the anterior development of the cranium was less than the posterior; yet the relation could not be called bad at his early age.

The boy is lively and seems intelligent, though his speech is imperfect, but he pronounced with facility after his father. He seemed unwilling to talk of his own accord before strangers; his father informs me, however, that he is very talkative at home and quite intelligent. His temper is good, and he is almost always in good-humor, but when excited by anger, his father alone can manage him, which he does by an old-fashioned, knock-down blow.

His father observed last night, when he slept with him for the first time, a constant erection of the penis, accompanied by a nickering, like an excited stallion, and for these reasons consulted me.

The boy has almost always slept by himself, and on an hard pallet on the floor. His back and shoulders are covered with the *acne simplex* of puberty. He has never been known to attempt masturbation, nor is it known whether he has had sexual relations, although the organ has that appearance. The slightest touch of the penis excites it, and the organ becomes tumid and of the average adult size, during the requisite examination.

The voice is that of puberty, and has been so for some time.

On the 15th of September, I visited him, accompanied by my friend and colleague, Professor John Frederick May, who verified the preceding examination and my measurements.

He is the seventh child and third son of his mother; weighed eleven and a half pounds at birth, and fifty-six pounds at three years.

At birth, the glans penis was perfectly uncovered, and the hair on the pubes half an inch long; at one year, things were just as they are now.

| | | | | | | |
|---|---|---|---|---|---|---|
| Around the thorax under axillæ, he measures | 2 | feet | $1\frac{1}{4}$ | inches. | | |
| " hips | " " | 2 | " | $2\frac{3}{4}$ | " | |
| " thigh (middle) | " " | 1 | " | 2 | " | |
| Penis in semi-flaccid state | " " | | | $4\frac{1}{4}$ | " | long. |
| " flaccid state | " " | | | $3\frac{3}{4}$ | inches *full* in circumference. | |

Around the arm, below insertion of the deltoid muscle, he measures eight inches.

Around the neck he measures one foot.

Around the head (above ears and over hair) he measures one foot eight inches.

From meatus auditorius to meatus of opposite side across the occiput, he measures $9\frac{3}{4}$ inches.

Although his neck is full, there is no remarkable development of the laryngeal cartilages, Pomum Adami.

The next question is in regard to the power of the testicles to secrete. Since I first saw this man-boy, his father has made inquiry as to this fact, and states the following to me as the result:

On the 13th of September, he slept with a near relative, a married lady, the mother of several children. In the middle of the night, she was aroused by finding the boy closely clasped to her back, and her night dress saturated. She thought he had emptied his bladder upon her, but on carrying her hand to the part, she found that it was saturated with a *very different and glutinous material* from that she expected.

I regret that I could not obtain the ejected matter to submit it to a microscopical test. The boy is extremely fond of embracing the opposite sex, though nothing further has been ascertained. In no other of the seven children borne by the same mother, has the same condition been observed, and in comparing an elder sister of ten years, I found she was extremely delicate, and only half an inch taller than Theodore.

I have several times seen him during an attack of nickering, and am satisfied that it is produced by a tendency to epilepsy.

Since writing this account I have been furnished by Dr. W. A. Williams, with a certificate, stating that he has known the family of Charles S—— for the last seven years; that he knows Theodore was born in September, 1848; that he saw Theodore when a very young infant, in the arms of his mother, and knows, from having seen him almost every week since that time, that he is the same child who was presented to the Pathological Society of the District of Columbia, by Dr. R. K. Stone, on Friday, Sept. 17, 1852.

Further, Dr. Williams states that he has attended the family professionally for about eighteen months, and was a student of medicine at the time

Theodore was born; and that he was first aware of his precocious sexual development in January, 1852, from actual inspection, though he had been informed of it at an earlier date.

In terminating this simple statement, I may observe that the father presented extreme precocity, having experienced his first sexual indulgence at the age of 8 years. He informed us that between the ages of 10 and 13 years he was a *better man* than he has ever been since. Delicacy forbids my detailing his prowess at that early age.

This extraordinary case will be perfectly under control for some time, and I will most willingly make any further observations which may be dictated by better heads.

It will be observed, that this is perhaps one of the most extraordinary cases on record, and I will now proceed to a hasty examination of a few of those to which reference has been made for me, in an exceedingly brief space of time, by my friend, Dr. R. D. Coolidge, U. S. A.

*Precocious Puberty in Males.*—1. An Account of a Child, 3 Years Old. By Gilbert Breschet, M. D. *Philadelphia Journ. of the Med. and Phys. Sciences*, 1821, vol. iii, pp. 417–18. James A. Sarin, born 20th October, 1817; 3 years and one month old at report; weighs fifty pounds, 3 feet 6¾ inches high; penis when flaccid, 4 inches long, and 5¼ when erect. Testicles *not enlarged* in proportion to penis.

2. *Good's Study of Medicine*, p. 73. N. Y. edition of 1829. Boiset, in the *Journal des Savans*, gives a case of a boy 3 years. *Philosophical Transactions*, 1745, boy 2 years 11 months. These were cases of great salacity, and no description given. Mr. Dankes, of St. Ives, near Huntingdon, reported the "Prodigium Willinghamense." The boy was buried at Willingham, and his epitaph was, "Born October 31, 1841, died September 3, 1747. At one year had signs of manhood, not 3 years was nearly 4 feet high; stupendous voice, and he died of premature old age."

3. Pliny (*Hist. Nat. Lib.* 8, c. 17,) reports a boy at Salamis 4 feet high, and attaining puberty at 3 years.

4. The case seen by Craterus, brother of Antigonus (*Phlegon, De Mirabilia*, c. 32,) who was infant, youth, adult, father, old man, and corpse, in 7 years.

5. *Milburger's Curiosities*, several cases.

6. Case reported by A. Lopez, M. D., of Mobile. *Amer. Journ. Med. Sciences*, vol. v, 1843, p. 500. Mulatto boy aged 3 years, 10 months, and 15 days; weight 82 pounds; height 4 feet half inch; width around chest 27½ inches; belly 27 inches; thigh 19 inches; arm 9½; circumference of head 22 inches; length of penis at rest 4; circumference 3½; his scrotum has a fair proportion to the other developments; but the *testes* have not descended; has whiskers; axillæ hairy; teeth 20, and *deciduous;* lifts a man of 140 pounds; covered with *acne simplex* (as Theodore S—— is) of puberty. Has spermatic odour, but not known whether he has venereal appetite; judge from stains on his shirt.

To these, I could add from *Beck's Med. Jurisp.*, p. 519, vol. i, 1838, several cases in both sexes.

Very many cases of precocious development in the female might be adduced: but to them I do not care to draw your attention; it is only necessary to recall the fact that the mother of a family on the banks of the Ganges need only be 9 years of age.

There has been no time to investigate this subject fully, but I think it will be difficult to find a case comparable to the one which I now present you. In most of the cases to which reference has been made, there is something wanting; and when we examine the totality of the appearances, there is not one, except the case described by Craterus, which approaches the proportion of Theodore S——.

WASHINGTON, D. C., Sept. 1852.—*Am. Jour. Med. Sciences.*

